# Geographical and Epidemiological Characteristics of 3,487 Confirmed Cases With COVID-19 Among Healthcare Workers in China

**DOI:** 10.3389/fpubh.2020.586736

**Published:** 2021-01-26

**Authors:** Maohui Feng, Zhixiao Li, Jun Xiong, Weiguo Xu, Boqi Xiang

**Affiliations:** ^1^Hubei Key Laboratory of Tumor Biological Behaviors, Department of Gastrointestinal Surgery, Wuhan Peritoneal Cancer Clinical Medical Research Center, Hubei Cancer Clinical Study Center, Zhongnan Hospital of Wuhan University, Wuhan, China; ^2^Department Anesthesiology and Pain Medicine, Tongji Hospital of Tongji Medical College, Huazhong University of Science and Technology, Wuhan, China; ^3^Hepatobiliary Surgery Center, Union Hospital, Tongji Medical College, Huazhong University of Science and Technology, Wuhan, China; ^4^Department of Orthopedics, Tongji Hospital of Tongji Medical College, Huazhong University of Science and Technology, Wuhan, China; ^5^Department of Psychology, University of California, Davis, Davis, CA, United States

**Keywords:** severe acute respiratory syndrome coronavirus 2, health workers, COVID-19 disease, human-to-human transmission, scientific protective measures

## Abstract

As the first area to report the outbreak, China used to be the front line of the battle against the novel coronavirus SARS-CoV-2. The present descriptive analysis of 3,487 COVID-19-confirmed cases with health workers reported through April 30, 2020 offers important new information to the international community on the epidemic in China. These data showed that Chinese measures including the high-grade protective gear used, mask wearing, and social distancing, are effective in reducing transmission in hospitals.

## Introduction

Severe acute respiratory syndrome coronavirus 2 (SARS-CoV-2) is a previously unknown virus that was first reported to cause severe respiratory infections in Wuhan, China, in late December 2019 (1–4). As the hardest-hit city in the outbreak, Wuhan city was shut down in January 23, 2020 to curb the rapid spread of the virus across China. Despite the local government's heightened disease control and prevention efforts, the highly contagious SARS-CoV-2 continued to spread, and some areas in Hubei province saw a spike in the number of infected cases.

Because of person-to-person transmission of the novel coronavirus SARS-CoV-2 (1, 5–12), healthcare workers were at an increased risk of infection as they were in close contact with patients infected by the coronavirus disease, COVID-19 (3). Some healthcare workers had contracted the COVID-19 on the front line of the battle against the new coronavirus. As of April 30, 2020, there were 3,487 confirmed COVID-19 cases of medical staff in China, including 2,961 (84.9%) in Wuhan city, one of the major epicenters of SARS-CoV-2 infections of medical staff in China. In this article, we reported geographical and epidemiological findings of 3,487 confirmed cases among healthcare workers in hospitals.

## Methods

We collected publicly available data from the Zi Jie Tiao Dong Humanitarian funding for medical workers infected by SARS-CoV-2, Red Cross Society of China, including batch 1–63 big data (https://it.gmw.cn/2020-03/30/content_33699059.htm?s=gmwreco2) and batch 64–70 between March 30 and May 8, 2020 (https://www.redcross.org.cn/html/2020-05/71073.html). All confirmed cases with COVID-19 were detected using viral nucleic acid testing. Analyses included the geo-temporal analysis, examination of age distributions and sex ratios, and department distributions of confirmed cases with healthcare workers.

## Results

### Epidemiological Curve of Confirmed Cases With Health Workers in Hospitals

**Figure 1:**
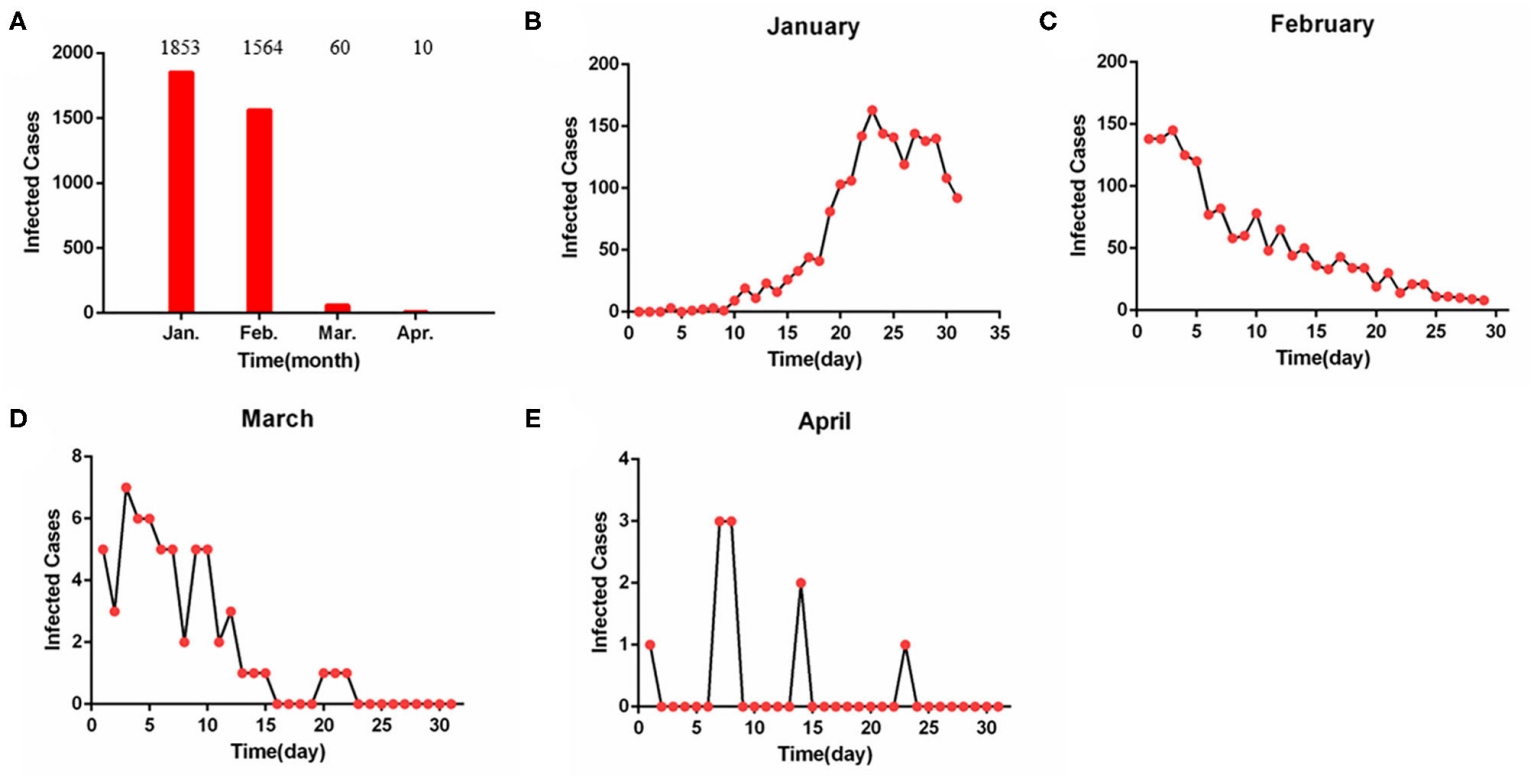
The epidemic curve of confirmed cases in health workers. **(A)** The confirmed cases from January to April. **(B)** The confirmed cases from January 1 to 30. **(C)** The confirmed cases from February 1 to 29. **(D)** The confirmed cases from March 1 to 30. **(E)** The confirmed cases from April 1 to 30. The confirmed cases peaked on January 23, then began to decline leading from February 2, 2020.

As of April 30, 2020, a total of 3,487 confirmed cases of healthcare workers were reported (Figure 1A). Among them, 1,853 cases (53.1%) were diagnosed before January 31, 2020 (Figure 1B); 1,564 cases (44.9%) were confirmed from February 1 to 29, 2020 (Figure 1C); 70 cases (2.0%) were infected from March 1 to April 30, 2020 (Figures 1D,E).

**Figure 2:**
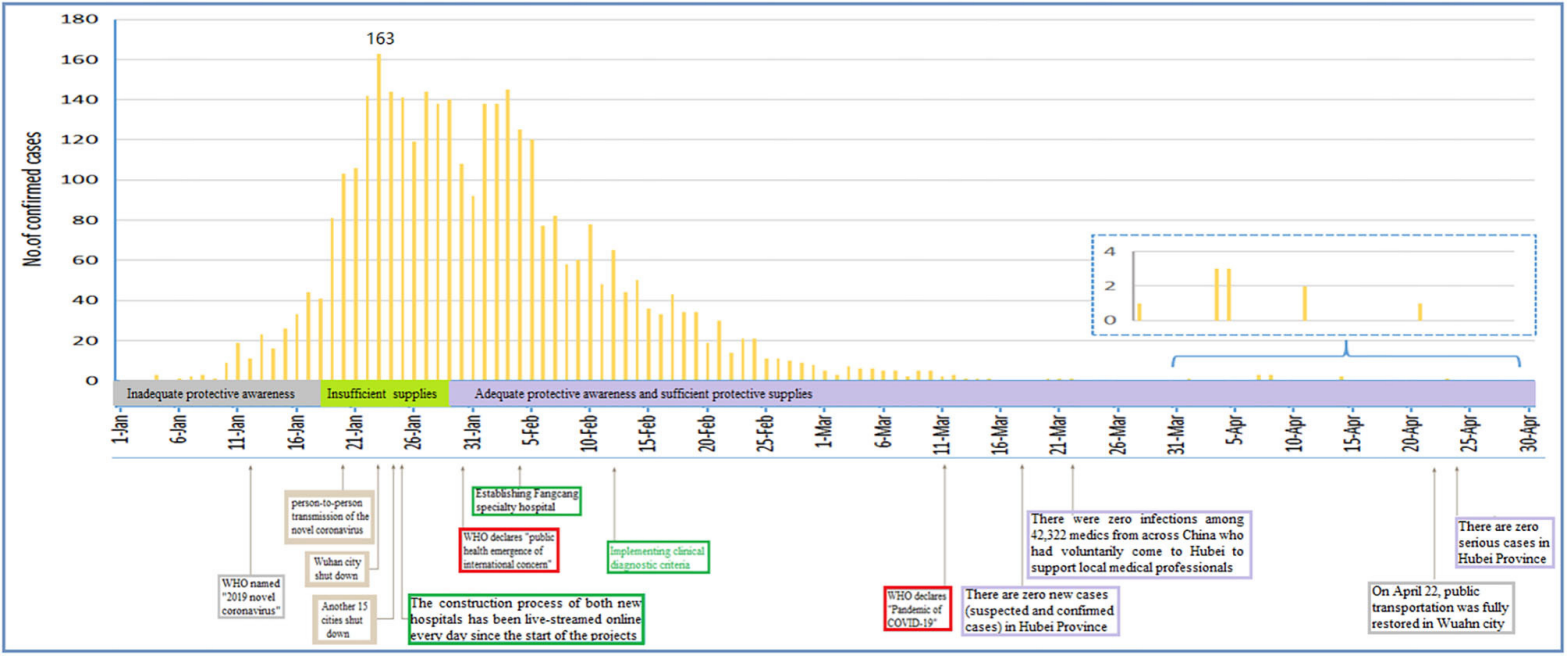
The graph's x-axis (dates from January 1 to April 30, 2020) is used as a timeline of the key events and dynamic profile of confirmed healthcare workers during the COVID-19 outbreak. The profiles of confirmed healthcare workers are shown in the graph's y-axis. The key international events included the international public health emergency (January 30, 2020) and the declaration of COVID-19 as a pandemic (March 11, 2020) by the WHO. The key national events included that Wuhan city shut down (January 23, 2020) and public transportation restored in Wuhan city (April 22, 2020). The statuses of “insufficient supplies” were determined based on insufficient high-grade mask wearing, e.p., the N95 masks, and insufficient high-grade protective gear was used. The statuses of “adequate protective awareness and sufficient protective supplies” were determined based on broadcast of critical information (e.g., promoting hand washing, mask wearing, and care seeking) with high frequency through multiple channels and mobilization of a multi-sector rapid response teams including 42,322 medical staff from across China who voluntarily came to Hubei to support local medical healthcare professionals from January 25 to March 26, 2020.

The COVID-19 epidemic curve with number of cases plotted by date of confirmed diagnosis of health workers from January 1, 2020 to April 30, 2020 is shown in Figure 2. Confirmed cases based on positive viral nucleic acid test were stacked to show the total daily cases. The inset showed that the peak number of confirmed cases for all cases overall occurred on January 23, 2020. Since February 2, 2020, confirmed cases of health workers had declined.

### Age Distribution and Sex Ratio

**Figure 3:**
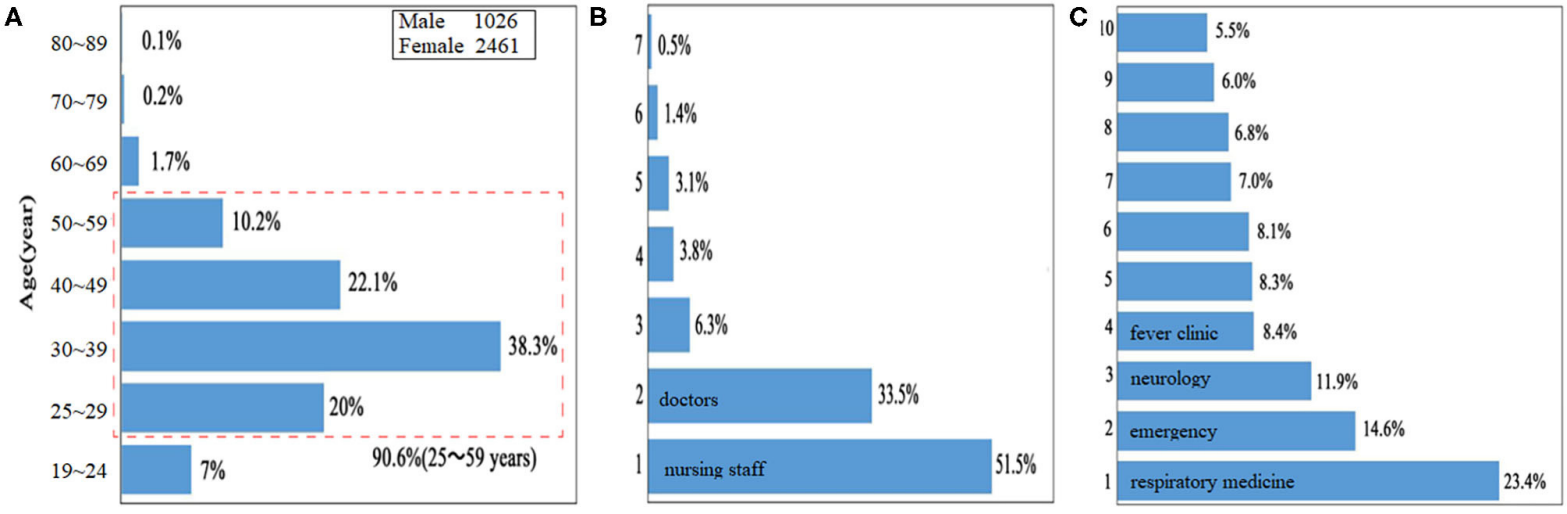
Clinical characteristics of confirmed healthcare workers. **(A)** Characteristics of age distributions. **(B)** Post distribution of confirmed healthcare workers, including nursing staff (51.5%), doctors (33.5%), administrative staff (6.3%), medical technicians (3.8%), logistics management staff (3.1%), pharmacists (1.4%), and others (0.5%); according to the number of confirmed cases with COVID-19, seven posts were arranged in sequence from code 1 to 7. **(C)** Top 10 distributions of confirmed healthcare workers in clinical departments, including the Department of Respiratory Medicine (23.4%), Emergency (14.6%), Neurology (11.9%), Fever Clinic (8.4%), Gynecology and Obstetrics (8.3%), Gastroenterology (8.1%), Critical Medicine (7.0%), Orthopedics (6.8%), Oncology (6.0%), and Cardiology (5.5%); according to the number of confirmed cases with COVID-19, 10 clinical departments were arranged in sequence from code 1 to 10.

The age distribution of cases in China overall is presented in Figure 3A. The proportion of confirmed cases 25–59 years of age at baseline (i.e., date of confirmed diagnosis) was 90.6% for cases in China overall (which includes Hubei Province and 14 other provincial-level administrative divisions). The male-to-female ratio (male, *n* = 1,026; female, *n* = 2,461) was 0.42:1 in China overall (Figure 3A).

### Post Distribution of Confirmed Healthcare Workers

Post distribution of confirmed healthcare workers is shown in Figure 3B. Among them, nursing staff (51.5%), doctors (33.5%), administrative staff (6.3%), medical technicians (3.8%), logistics management staff (3.1%), pharmacists (1.4%), and others (0.5%) were diagnosed; according to the number of confirmed cases with COVID-19, 7 posts were arranged in sequence from code 1 to 7.

### Top 10 Distributions of Confirmed Healthcare Workers in Clinical Departments

Top 10 distributions of confirmed healthcare workers in clinical department are shown in Figure 3C. Among them, the Department of Respiratory Medicine (23.4%), Emergency (14.6%), Neurology (11.9%), Fever Clinic (8.4%), Gynecology and Obstetrics (8.3%), Gastroenterology (8.1%), Critical Medicine (7.0%), Orthopedics (6.8%), Oncology (6.0%), and Cardiology (5.5%) were identified; according to the number of confirmed cases with COVID-19, 10 clinical departments were arranged in sequence from code 1 to 10.

### Geographical Epidemiological Characteristics of 10 Hospitals With Confirmed Cases in Wuhan

**Figure 4:**
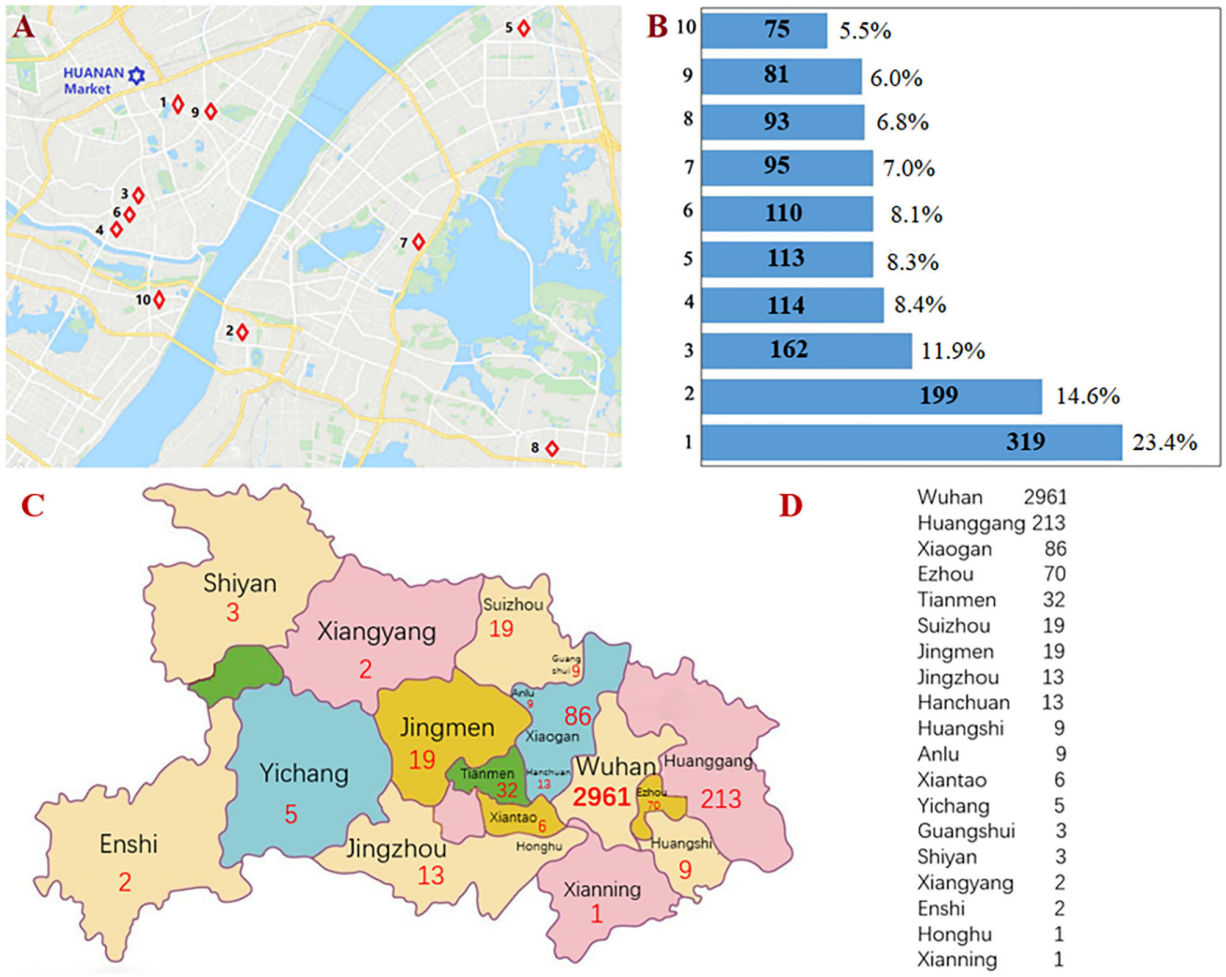
Geographical epidemiological characteristics of 10 hospitals and 17 counties with confirmed healthcare workers. **(A)** Geographical distribution of 10 hospitals in Wuhan city. **(B)** According to the number of confirmed cases with COVID-19, 10 hospitals were arranged in sequence from code 1 to 10. **(C)** Geographical distribution of 19 cities in Hubei Province. **(D)** According to the number of confirmed cases with COVID-19, 17 counties were arranged in sequence from code 1 to 17.

By April 30, 2020, 10 hospitals in Wuhan had reported over 1,361 confirmed cases among medical staff. Geographical distribution of these hospitals is shown in Figure 4A. According to the number of confirmed case with COVID-19, these hospitals were arranged in sequence as code 1–10. Hospital-1 and−9 are closer to the Huanan Seafood Wholesale Market than the rest, and reported 319 and 81 COVID-19-confirmed cases of medical staff, respectively (Figure 4B). Four hospitals farther from the Huanan Seafood Wholesale Market are located in the south region of the Yangtze River and reported 199, 113, 95, and 93 COVID-19-infected cases, respectively (Figure 4B).

### Geographical and Epidemiological Characteristics of 17 Counties With Confirmed Healthcare Workers in Hubei Province

As of April 30, 2020, a total 3,487 confirmed cases were diagnosed from 15 provinces, autonomous regions, and municipalities, and Hubei Province (3,441 cases) accounted for 98.7%. Huanggang, Xiaogan, and Ezhou counties were closer to Wuhan city than the rest, and reported 213, 82, and 70 COVID-19-confirmed cases of medical staff, respectively (Figures 4C,D).

## Discussion

A main finding of this characterization analysis among healthcare workers infected with COVID-19 was that this novel coronavirus is highly contagious. Here we offered a first description of the 3,487 confirmed cases among health workers between first recognition of the outbreak of unknown pneumonia on December 31, 2019 to the end of the study period on April 30, 2020. Even with extreme response measures in Wuhan and another 15 cities including the complete shutdown and isolation of whole cities on January 23, 2020, cancellation of Chinese New Year celebrations, and prohibition of attendance at school and work, the coronavirus continued to spread rapidly, and some areas saw a spike in the number of infected healthcare workers.

A major contribution of the current study was a first description of the epidemic curves for COVID-19-confirmed cases with health workers. Figures 1, 2 showed the COVID-19 epidemic curve with the number of cases plotted by confirmed date from January 1, 2020 to April 30, 2020 for all cases among health workers nationwide. Our data showed that peak timing of confirmed date among infected cases occurred on January 23, 2020. In addition, confirmed cases with health workers had declined after February 3, 2020 (Figure 2). Data from The Novel Coronavirus Pneumonia Emergency Response Epidemiology Team reported subgroup analysis of all cases among health workers including confirmed vs. suspected, clinically diagnosed, and asymptomatic cases, and indicated that a total of 3,019 health workers had been infected (1,716 confirmed cases) in the 422 medical facilities serving COVID-19 patients (13).

The status “adequate protective awareness and sufficient protective supplies” means high-grade protection for the healthcare workers in a way in Figure 2. This status covered from January 30 to April 30. However, there were still a lot of infected cases after the adequate protective awareness and sufficient protective supplies, and a peak on February 2 after a decrease on January 30. The reasons for this were that (1) because of the incubation period (7–14 days) of the COVID-19, some healthcare workers were infected with SARS-CoV-2 from January 15 to 29, 2020 (period of insufficient protective supplies), and they were confirmed with SARS-CoV-2 after January 30, 2020 (period of sufficient protective supplies). (2) A large proportion of cases were not confirmed by nucleic acid testing from January 15 to January 29, 2020, since this process is slow, labor intensive, and requires specialized equipment and skilled technicians.

In light of this rapid spread, early assessment was that the virus might be from a still-unknown animal into humans at the Huanan Seafood Wholesale Market in Wuhan (2, 14, 15). A new cluster of 114 COVID-19 cases in Beijing on June 11–16, 2020 had been traced to the sprawling Xinfadi seafood market (16). The common characteristics of the two seafood markets are important to understand the origin of the novel coronavirus SARS-CoV-2 and links between cases.

Huang et al. (2) showed 41 patients with COVID-19 cases who had a history of exposure to the Huanan Seafood Wholesale Market. Thus, we described the geographical epidemiological characteristics for 10 hospitals with infected healthcare workers by using Figure 4A. On April 30, 2020, there were 319 and 122 COVID-19-confirmed cases of medical staff in Hospital-1 and−9, which were closer to the Huanan Seafood Wholesale Market, respectively. The number of cases in these two hospitals (Hospital-1 and−9) are largely different; the reason for this was that at the early days of the outbreak, the administrators of Hospital-1 did not encourage mask wearing and care seeking, e.g., using protective gear, resulting in a lot of infected cases for the healthcare workers including four dead cases, whereas the administrators of Hospital-9 promoted mask wearing and care seeking.

Social distancing is very important to curb the epidemic of the COVID-19 (17). In accordance with the principle of putting the safety of the masses and health first, the government authorities had adopted maximum effort and scientific measures to curb the spread of the outbreak. With the exception of the residents under quarantine indoors for 14 days, local authorities had stepped up disinfection, ventilation, and screening measures in public spaces, and got manufacturers of protective suits, surgical masks, safety goggles, negative pressure ambulances, and drugs back in full production as soon as possible. Beginning on January 23, 2019, health and public health personnel as well as military medical units were massively mobilized and voluntarily came to Wuhan city. As of March 22, 2020, a total of 42,322 medical staff from across China supported local medical healthcare professionals (Figure 2). Despite the extremely rapid spread of the novel coronavirus (18–22), there was zero infection among the 42,322 health workers, suggesting that China's coronavirus response highlights the importance of implementing effective public health strategies.

In conclusion, the present descriptive analysis of 3,487 COVID-19-confirmed cases with health workers reported through April 30, 2020 offers important new information to the international community on the epidemic in China. Despite this analysis chronicles the extremely rapid spread of the novel coronavirus, Chinese scientific measures including high-grade protection and social distancing are crucial strategies to prevent human-to-human transmission in hospitals.

## Data Availability Statement

The original contributions presented in the study are included in the article/supplementary materials, further inquiries can be directed to the corresponding author/s.

## Author Contributions

MF, ZL, and JX: data collection. WX and BX: data interpretation and writing. All authors contributed to the article and approved the submitted version.

## Conflict of Interest

The authors declare that the research was conducted in the absence of any commercial or financial relationships that could be construed as a potential conflict of interest.
